# Bone Cutting Forceps: A Safe Approach for Saving Strangulated Penis

**DOI:** 10.1155/2016/1274124

**Published:** 2016-04-28

**Authors:** Mohamed Ahmed Abd El Salam, Ahmed Gamal, Hossam Elenany

**Affiliations:** Faculty of Medicine, Cairo University, Kasr Al Ainy Street, Cairo 11562, Egypt

## Abstract

Penile strangulation is considered a rare andrological emergency that may lead to a wide range of complications. Penile strangulation may be related to mental retardation in children or hypersexuality, abnormal sexual preferences in adults. This case report shows a 23-year-old male who presented to the emergency room with agonizing pain related to a metallic ring placed at the base of the penis for more than 8 hours during an attempt of masturbation. Removing this metallic ring and saving the patient's penis from gangrene without causing injury were very challenging. Patient was successfully managed and had an uneventful recovery.

## 1. Introduction

Strangulation of the penis is a rare emergency presentation in daily practice that requires urgent intervention to avoid possible complications. Strangulation may be caused by a wide variety of objects that could be metallic [1] or nonmetallic [2] and are usually associated with an attempt to improve sexual pleasure and/or to maintain a longer period of erection.

Strangulation of the penis by an encircling object leads to swelling of the penis distal to the object because of the initial blockage of the venous return and arterial blood supply. After several hours, penile strangulation can result in ischemic necrosis and gangrene of the tissues. On the other hand, if the encircling object blocks the venous return without any arterial involvement, it may result in massive enlargement of the penis due to lymphedema. In the latter, the necrosis may result from anoxia associated with venous stasis.

In addition, a whole spectrum of various degrees of mechanical penile injuries is recognized such as skin ulceration, urethral injuries, constriction of corpus spongiosum and corpora cavernosa, development of urethral fistula, and loss of distal penile sensations [3].

## 2. Case Presentation

A 23-year-old married male patient presented to the emergency department in our hospital (Kasr Al Ainy) complaining of severe agonizing penile pain and swelling for more than 8 hours following placement of a metallic ring at the base of the penis while attempting at increasing his sexual arousal and pleasure which finally ended by being stuck and inability to pass urine.

**Figure 1:**
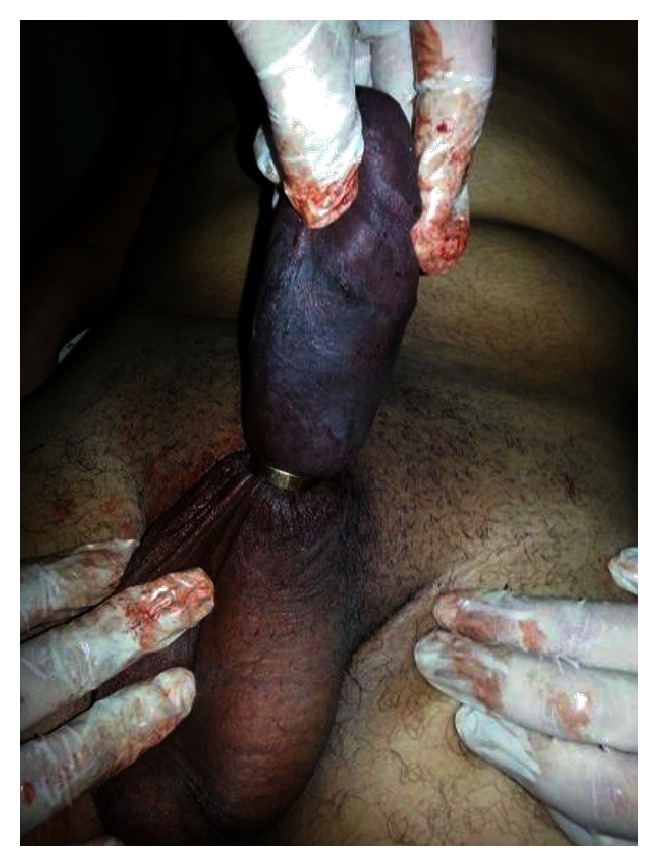
Inside the operative theatre, severe penile edema and color change due to the tight metallic ring at the base of the penis.

On clinical examination the vital signs were normal but the penis was swollen, tender, edematous, cold, and bluish in color with poor capillary refilling with attenuated distal penile pulsations and sensations. Also, there was a metallic ring stuck at the root of the penis (Figure 1).

**Figure 2:**
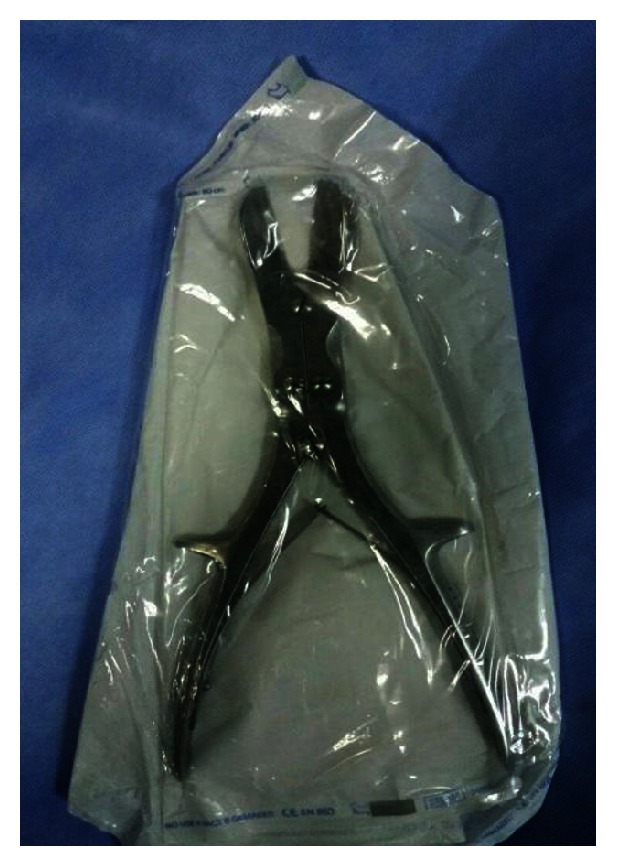
Bone cutting forceps used.

Attempts to remove the ring with lubricant gel and compression were done but we did not succeed because the ring was too tight due to massive penile swelling. After that, the patient was admitted to the operating theatre and penile block was done. Each penile nerve was blocked separately at the level of the penile root using bupivacaine (without adrenaline) in concentration of 0.25%. Attempts to release the penis were done either manually or with the usage of the surgical equipment but unfortunately this was not possible. The metallic ring was cut from one side by a bone cutting forceps (Stille Liston Bone Cutting Forceps 23 cm Curved Orthopedic Surgical Instruments) (Figure 2), the blunt surface of the forceps blade was introduced tangentially between the ring and the penis with the help of lubricant gel, and then rotation of the forceps was done to get the metallic ring between its sharp blades and the penis was liberated; afterwards the patient was under observation for 48 hours, administration of antibiotics (amoxicillin/clavulanic acid 625 mg twice daily for 5 days to avoid infection as the tissues were edematous and there were minor skin abrasions from the bone cutting forceps usage) and antiedematous measures (trypsin 5 mg + chymotrypsin 5 mg) used 3 times daily for 1 week, in addition to penile elevation.

**Figure 3:**
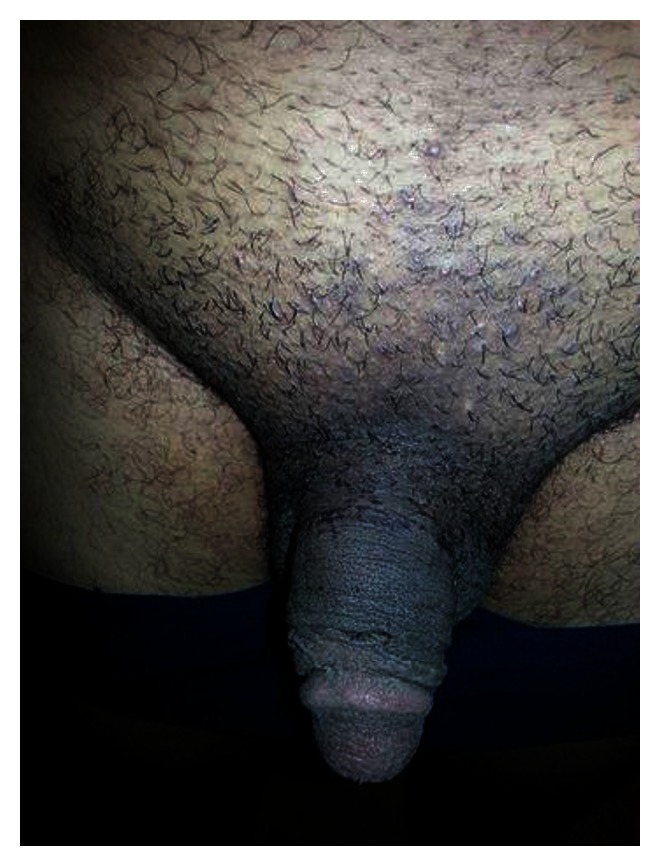
After 2 weeks of the admission.

The patient was able to pass urine immediately after penile release; twenty-four hours later the distal penile pulsations, sensations, and capillary refill were regained with partial subsidence of the edema. The patient regained his complete erectile capacity during his follow-up (Figure 3).

## 3. Discussion

Penile strangulation is an unusual clinical condition reported for the first time in 1755 by Gauthier [4]. Since then, approximately 60 cases have been reported in the world literature. No standardization for the penile salvage was found superior with each case managed individually according to its clinical findings and operative settings [5].

Minor penile injuries may lead to a series of physical and psychological impacts. Some conditions such as priapism, paraphimosis, and penile strangulation may lead to interruption of the blood supply of the penis and if not managed as early as possible may lead to ischemia, affection of the erectile capacity, necrosis, and subsequent venous gangrene of the penis [6]. So early management avoids possible spectrum of complications.

Gangrene is an uncommon outcome, as each corpus cavernosum has an individual artery (cavernosal artery), thick Buck's fascia, tough tunica albuginea, and compressible corporeal tissue resisting pressure on the deep vessels [7].

Depending on the nature of the constricting object, whether metallic or nonmetallic, pressure on the tissues, and the duration of strangulation, the prognosis and outcome is variable. Bhat et al. on 1991 [3] have graded the penile strangulation injuries as follows:

- Grade I: edema of distal penis; no evidence of skin ulceration or urethral injury.
- Grade II: injury to skin and constriction of corpus spongiosum, but no evidence of urethral injury; distal penile edema with decreased penile sensation.
- Grade III: injury to skin and urethra but no urethral fistula; loss of distal penile sensations.
- Grade IV: complete division of corpus spongiosum leading to urethral fistula and constriction of corpora cavernosa with loss of distal penile sensations.
- Grade V: gangrene, necrosis, or complete amputation of distal penis.

The choice of method for removal depends upon type, size, incarceration time, trauma grade, and availability of the equipment. There is no standard protocol mentioned to deal with such cases. Every case needs an individual approach depending upon the circumstances and facilities available [8].

Treatment techniques for penile strangulations can generally be divided into four groups: the string technique and its variants, with and without aspiration of blood from the glans; aspiration techniques; cutting devices; and surgery [9].

Our case had grade II penile strangulation and we used a bone cutting forceps to cut the metallic ring under penile block that was done successfully with favorable outcomes in the follow-up visits with no erectile function affection and complete regaining of the morning erections and sexual functions.

In conclusion, penile strangulation is a rare clinical condition and the consequences are broad, ranging from mild nonsignificant neurovascular compromise that resolves after decompression to severe gangrene of the penis. So, early diagnosis and proper, rapid management of such cases could lead to favorable prognosis and better outcome.
